# Generation of Leukemia Inhibitory Factor-Dependent Induced Pluripotent Stem Cells from the Massachusetts General Hospital Miniature Pig

**DOI:** 10.1155/2013/140639

**Published:** 2013-11-26

**Authors:** Dae-Jin Kwon, Hyelena Jeon, Keon Bong Oh, Sun-A Ock, Gi-Sun Im, Sung-Soo Lee, Seok Ki Im, Jeong-Woong Lee, Sung-Jong Oh, Jin-Ki Park, Seongsoo Hwang

**Affiliations:** ^1^Animal Biotechnology Division, National Institute of Animal Science, RDA, Suwon, Gyeonggi 441-706, Republic of Korea; ^2^Research Center of Integrative Cellulomics, Korea Research Institute of Bioscience and Biotechnology, Daejeon 305-806, Republic of Korea; ^3^Department of Animal Biotechnology, Jeju National University, Jeju 690-756, Republic of Korea

## Abstract

The generation and application of porcine induced pluripotent stem cells (iPSCs) may enable the testing for safety and efficacy of therapy in the field of human regenerative medicine. Here, the generation of iPSCs from the Massachusetts General Hospital miniature pig (MGH minipig) established for organ transplantation studies is reported. Fibroblasts were isolated from the skin of the ear of a 10-day-old MGH minipig and transduced with a cocktail of six human factors: *POU5F1, NANOG, SOX2, C-MYC, KLF4,* and *LIN28*. Two distinct types of iPSCs were generated that were positive for alkaline phosphatase activity, as well as the classical pluripotency markers: *Oct4, Nanog, Sox2*, and the surface marker Ssea-1. Only one of two porcine iPSC lines differentiated into three germ layers both *in vitro* and *in vivo*. Western blot analysis showed that the porcine iPSCs were dependent on LIF or BMP-4 to sustain self-renewal and pluripotency. In conclusion, the results showed that human pluripotent factors could reprogram porcine ear fibroblasts into the pluripotent state. These cells may provide a useful source of cells that could be used for the treatment of degenerative and genetic diseases and agricultural research and application.

## 1. Introduction 

 Porcine pluripotent stem cells (PSCs) are important for modeling embryonic development and disease processes in biomedical research; they are especially important in transplantation medicine, immunology, and the study of the circulatory system [1, 2]. For these reasons, the development of porcine induced pluripotent stem cells (iPSCs) could be valuable to study the characteristics of porcine PSCs and develop clinical models that might be applied to human disease. However, despite years of effort, the pluripotency of porcine PSCs has not yet been clearly demonstrated.

 However, recently, iPSCs have been successfully generated by reprogramming somatic cells using defined transcription factors [3]; several studies have reported the derivation of porcine iPSCs from fibroblasts using a common combination of reprogramming factors [4–6]. These initial reports indicate that iPSCs generated from pigs are more similar to human PSCs than mouse PSCs with regard to their general morphology, pluripotent marker expression, and signaling dependence. There are two distinct categories of PSC characteristics [7]. In the naïve state of PSCs that corresponds to the preimplantation of the inner cell mass, the cells are characterized by compact, dome-like colonies and inactivation of the X chromosomes in the female cell lines [8–10]. Cytophysiologically, they are dependent on LIF/STAT3 signaling for maintenance of the undifferentiated state and bone morphogenetic protein 4 (BMP-4) for self-renewal and resistance to differentiation [11, 12]. These cells have the capacity to differentiate into three germ layers (ectoderm, mesoderm, and endoderm) both *in vitro* and *in vivo* and can contribute to the development of chimeras when injected into allogenic embryos, which result in germline chimeric offspring [13, 14]. In addition, there is the primed pluripotent state of stem cells, derived from the postimplantation phase epiblasts and referred to as epiblast stem cells (EpiSCs); these cells show a flattened colony morphology and a dependence on FGF and activin signaling for maintenance of pluripotency and self-renewal [15, 16]. The major difference between the two pluripotent stem cell states lies in their ability to develop chimeric offspring. Although the primed pluripotent state stem cells can differentiate into all three germ layers *in vitro*, they cannot develop into chimeras [16, 17]. Therefore, establishing the naïve state porcine PSCs would provide a greater opportunity to develop large scale applications of these stem cells to biomedical research and agriculture. 

The results of this study show that porcine iPSCs that were directly generated from ear skin fibroblasts, using lentiviral vector expressing human factors: *POU5F1 (OCT4), NANOG, SOX2, C-MYC, KLF4,* and *LIN28*, generated porcine iPSCs that had characteristics similar to those of naïve-like pluripotent stem cells and maintained their pluripotency and self-renewal by the LIF or BMP-4 mediated pathway. 

## 2. Materials and Methods

### 2.1. Somatic Cell Culture

 Porcine ear fibroblasts (PEFs) were derived from a 10-day-old Massachusetts General Hospital (MGH) Major Histocompatibility Complex (MHC) inbred miniature pig (MGH pig) [18]. The ear tissue was chopped into small pieces and then enzymatically digested with 0.5% trypsin-EDTA (GIBCO) in PBS (GIBCO) for 30 min at 37°C. The digested tissues were cultured with basic medium consisting of Dulbecco's modified Eagle's medium (DMEM/F12; GIBCO), 10% ES cell fetal bovine serum (GIBCO), 50 units/mL penicillin (GIBCO), 50 *μ*g/mL streptomycin (GIBCO), 2 mM L-glutamine (GIBCO), and 1 uM *β*-mercaptoethanol (Sigma) at 37°C with 5% CO_2_. The PEFs were cultured up to a confluency and either passaged with a 1 : 2 division or stored in liquid nitrogen for further experiments. 

### 2.2. Lentiviral Transduction and Culture

 Lentiviral transduction was performed using the viPS Vector Kit (Thermo Fisher Scientific) following the manufacturer's instructions. The PEFs were plated at a density of 1.5 × 10^4^ cells/cm^2^ in 4-well culture dishes and cultured with basic media containing 2 mM valproic acid (Sigma) for 24 h before transduction. The PEFs were then transduced with lentiviral vectors encoding six human transcription factors (*POU5F1, NANOG, SOX2, C-MYC, KLF4,* and *LIN28*) to initiate reprogramming via ectopic expression. After 24 h of transduction, the PEFs were harvested and plated onto mitomycin C inactivated mouse embryonic fibroblasts (iMEFs) in stem cell medium, which is composed of DMEM/F12 culture medium supplemented with 10% Knockout Serum Replacement (KSR; Invitrogen), 10% FBS (Invitrogen), 50 units/mL penicillin (GIBCO), 50 *μ*g/mL streptomycin (GIBCO), 2 mM L-glutamine (GIBCO), 0.1 mM nonessential amino acids (NEAAs, GIBCO), 1 uM *β*-mercaptoethanol, 20 ng/mL basic fibroblast growth factor-2 (bFGF; R&D Systems), and 20 ng/mL leukemia inhibitory factor (LIF; Sigma). When the colonies were grown large enough to isolate, they were cut into small pieces using a hook under a dissection microscope and passaged into 35 mm dishes with stem cell medium without bFGF. Porcine iPSCs were maintained by manual passage every 4-5 days. For the population doubling time (PDT) and karyotype analysis, porcine iPSCs were transferred to feeder-free conditions by trypsinization onto growth-factor-reduced Matrigel (diluted 1 : 100 in DMEM/F12; BD Biosciences) coated plates in stem cell media and further passaged every 3-4 days (~80% confluence). The PDT was estimated by counting the cell number at the time of passage and calculated using the log⁡_10_(*N*/*N*_0_) × 3.33 formula (where *N* is the number of cells harvested and *N*_0_ is the number of cells plated) [19]. Karyotype analysis was performed after 15 passages using a standard high-resolution G-banding method at GenDix (http://www.gendix.com/). To examine the cytokine dependency of the porcine iPSCs, cells were cultured in the presence of 20 ng/mL LIF, 20 ng/mL bFGF, 20 ng/mL BMP-4 (Prospec), 20 ng/mL LIF + 2 *μ*M SU5402 (SU; Sigma), or 20 ng/mL bFGF + 1 *μ*M JAK Inhibitor I (JAKi; Santa cruz) for 48 h.

### 2.3. Alkaline Phosphatase (AP) Staining and Immunocytochemistry

 Porcine iPSCs were fixed with 4% paraformaldehyde for 20 min at room temperature and then washed two times with phosphate-buffered saline (PBS). AP staining was performed using the Vector Red Alkaline Phosphatase Substrate Kit I (VECTOR Laboratories, CA, USA) according to the manufacturer's protocol. The cells were incubated with substrate solution at room temperature until suitable staining developed. The cells were observed with the Leica Microsystem (Switzerland) and captured by the Leica Application Suite (ver 3.8.0) program. For immunocytochemistry, the fixed cells were incubated in blocking buffer containing 6% horse serum (Invitrogen) and 0.1% Triton X-100 (Sigma) for 40 min at RT. The cells were then cultured with primary antibodies diluted in the blocking buffer for 1 h at RT. Primary antibodies, Oct4 (1 : 100; Santa Cruz), Sox2 (1 : 100; R&D Systems), Nanog (1 : 100; Abcam), and Ssea-1 (1 : 100; R&D Systems), were detected by Alexa Fluor 488 (Invitrogen) conjugated secondary antibodies. All images were obtained by sequential scanning of the sample using the LSM 510 Meta NLO microscope (Zeiss, Jena, Germany) and merged with the Zeiss LSM image browser (ver. 3.2.0.70).

### 2.4. *In Vitro* and *In Vivo* Differentiation

*In vitro* differentiation was determined by embryoid body (EB) formation. EBs were produced using the AggreWell plate (Stemcell Technologies) following the manufacturer's instructions. The aggregated cells were then transferred to a Petri dish (BD Falcon) suspension culture in stem cell medium without LIF, and the medium was changed every other day for 10 days. The *in vivo* differentiation assay was performed using the teratoma formation test. Porcine iPSCs were harvested and 0.5~1 × 10^7^ cells in 0.2 mL volume with 30% Matrigel solution were injected subcutaneously into a nude mouse (Nara biotech). After 9 weeks, teratomas were dissected and fixed in 10% (v/v) neutral buffered formalin. Paraffin embedded samples were dissected and stained with hematoxylin and eosin following standard procedures for histological analysis. The teratoma sections were viewed with Leica Microsystems (Switzerland) and captured by Leica Application Suite (ver 3.8.0) program.

### 2.5. Reverse Transcription and Quantitative PCR

For the reverse transcription polymerase chain reaction (RT-PCR) analysis, RNA and DNA were extracted using RNeasy plus mini kits (Qiagen) and the DNA Blood DNeasy Kit (Qiagen) following the manufacturer's protocols, respectively. The total RNA and DNA concentrations were measured using the NanoDrop 1000 spectrophotometer (Thermo Scientific). RNA was reverse transcribed using a High-Capacity cDNA Reverse Transcription Kit (Applied Biosystems) following the manufacturer's protocol. PCR amplification was performed using GoTaq Green (Promega). PCR reactions were performed by initially denaturing cDNA at 95°C for 3 min followed by 35 cycles of denaturing at 95°C for 60 sec, annealing at a temperature specific for each primer set for 30 sec, polymerization at 72°C for 30 sec, and a final 10 min extension. PCR products were loaded into 2% agarose gels containing 0.6 mg/mL ethidium bromide and run in Tris-acetate-ethylenediaminetetraacetic acid buffer for 45 min. The gel documentation station was used to assess the PCR products (E-Graph AE-9000, ATTO).

Quantitative PCR was performed using the Rotor-Gene SYBR Green PCR Kit (Qiagen) on the Roter-Gene 6000 (Corbett Research). The conditions for real-time RT-PCR were as follows: 95°C, 5 min, followed by 35 amplification cycles (95°C, 5 sec; 60°C, 10 sec). The reaction was terminated by an elongation and a data acquisition step at 72°C for 30 sec. The expression value of each gene was normalized to the amount of *glyceraldehyde-3-phosphate dehydrogenase* (*Gapdh*), cDNA and the relative expression ratio of target genes was calculated by the ΔΔCt method. The primer sets for PCR analysis are listed in Table 1. 

### 2.6. Western Blotting

Proteins were extracted using a mammalian protein extract reagent supplemented with a protease inhibitor cocktail (Roche) as per the manufacturer's protocol. The proteins were quantified using the Bradford assay (Bio-Red, CA, USA). Then, the proteins were loaded on a 10% sodium dodecyl sulfate polyacrylamide gel (Bio-Red) and subjected to SDS-PAGE; the separated proteins were then transferred onto membranes and blotted onto polyvinylidene fluoride membranes (Invitrogen). The primary antibodies were used against SMAD 1/5/8, phospho-SMAD 1/5/8, Stat3, phospho-Stat3, and beta-actin, which were used with the appropriate HRP-conjugated secondary antibodies. Protein expression was detected using the ECL chemiluminescence Kit for Western blot analysis (GE Healthcare, Buckinghamshire, UK) according to the manufacturer's protocol; the signals were quantitated using the ImageJ (National Institutes of Health, Bethesda, MD).

### 2.7. Statistical Analysis

At least three replicates were performed for each treatment. PDT and real-time data were analyzed with Duncan's multiple range tests, using the general linear model procedure in SAS (SAS Institute, Inc., Cary, NC, USA). A probability of *P* < 0.05 was considered significant. 

## 3. Results

### 3.1. Generation of Porcine iPSCs

**Figure 1:**
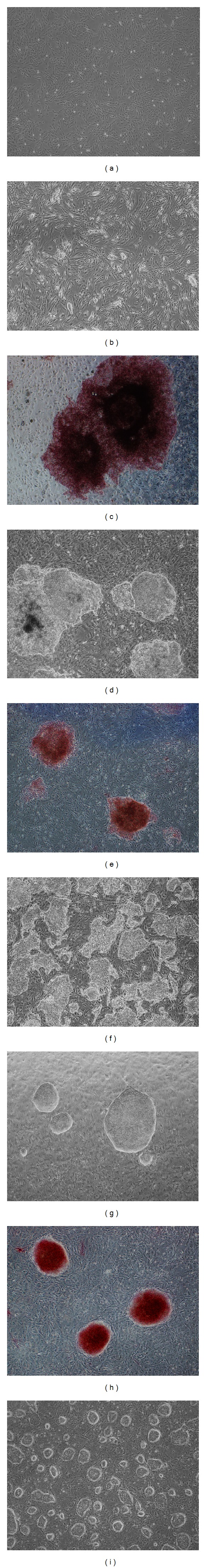
Generation of iPSCs from porcine ear fibroblast cells. Phase contrast images of PEFs (a) and being reprogrammed cells after 4 days of lentiviral transduction (b). The reprogrammed cells formed colony-like structures after first passage, which were positive for alkaline phosphatase (c). Two lines, A10 at passage 10 (d) and A15 at passage 8 (g), were maintained for the further experiments and showed AP activity ((e) and (h)). Both lines were cultured at a feeder-free condition and then replated onto iMEF feeder. A10 exhibited a flat morphology (f) while A15 formed a dome-shaped colonies with a sharp border (i).

PEFs from the MGH minipig were successfully reprogrammed using six human factors: *POU5F1, KLF4, NANOG, SOX2, C-MYC,* and *LIN28*. The transduced cells were cultured onto iMEF with 10% FBS/KSR stem cell medium supplemented with bFGF and LIF (Figure 1(a)). Four days after transduction, the transduced cells showed a high nuclear to cytoplasm ratio and prominent nucleoli, but they did not form compact colonies when they were further cultured (Figure 1(b)). Eighteen days after transduction, compact colonies emerged that were positive for AP (Figure 1(c)). Two of them were selected for further culture and analysis. One was A15, which grew rapidly as compact, tight colonies with a dome-shaped appearance (Figure 1(g)) and typical mouse ESC characteristics. The other was A10, which exhibited a flat and tightly packed morphology with sharp edges, and the cells had a high nucleus/cytoplasm ratio and prominent nucleoli, similar to human ESCs (Figure 1(d)). AP activity is one of the main characteristics of pluripotency. A15 line showed strong AP activity (Figure 1(h)), but the A10 line was only partially stained with AP (Figure 1(e)). Some of the colonies in the A10 line exhibited very little or no AP activity. Both porcine iPSCs were routinely passaged on feeder systems every three to four days without altering their characteristics. Porcine iPSCs cultured with a feeder-free system, Matrigel coated plates, and stem cell medium supplemented with 20 ng/mL LIF were positive for alkaline phosphatase (data not shown). Furthermore, porcine iPSCs from these conditions were placed into MEF feeder conditions, and there was no visible differentiation in addition to those that were maintained in the traditional feeder system (Figures 1(f) and 1(i)). During expansion in culture, the iPSCs colonies retained a compact undifferentiated morphology. 

### 3.2. Characterization of Porcine iPSCs

**Figure 2:**
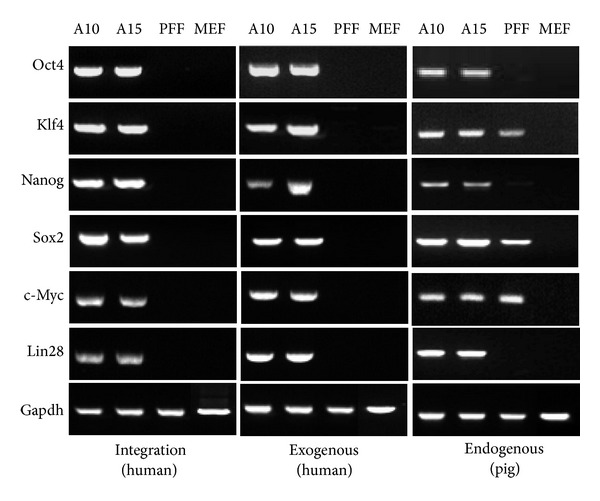
Integration of transgene and pluripotent gene expression in porcine iPSCs. Genomic PCR was conducted to confirm the integration of transcription factors, while exogenous or endogenous gene expression was identified using RT-PCR. Both two lines had integrated all six transcription factors, which were expressed exogenously in further culture. Furthermore, all six pluripotent genes were endogenously expressed in both A10 and A15 at passage 20.

**Figure 3:**
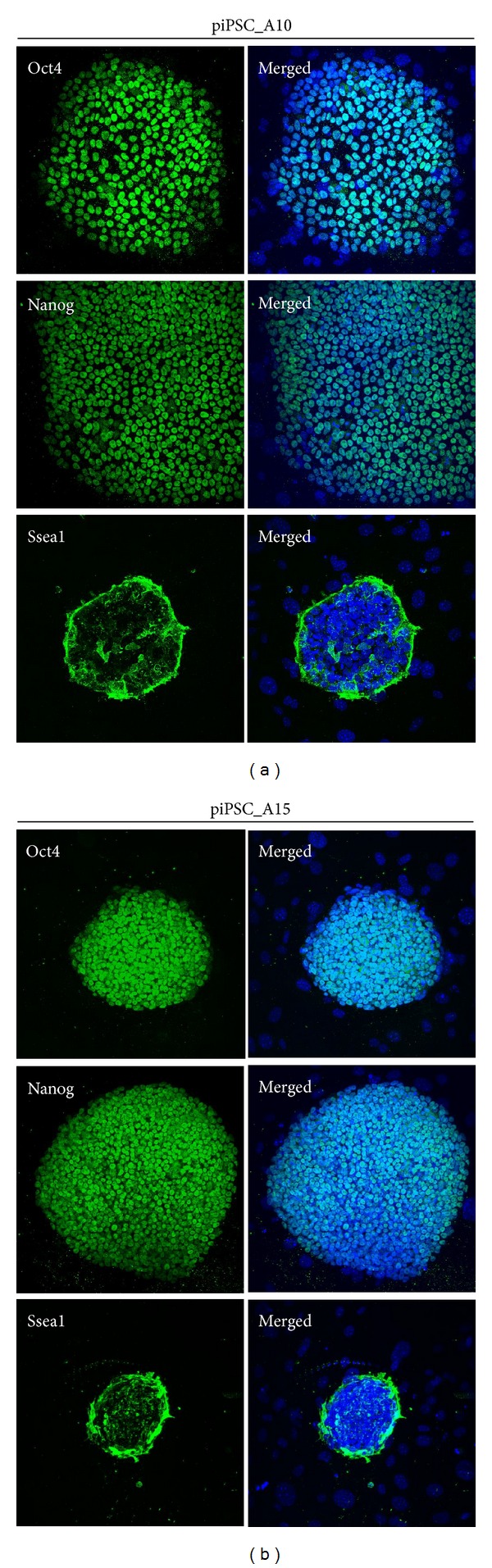
The expression of pluripotent markers in porcine iPSCs. Immunocytochemistry shows that both two lines, A10 (a) and A15 (b), express Oct4, Nanog, and surface maker Ssea1. However, other surface makers Ssea4, Tra-1-60, and Tra-1-81 are not expressed (data not shown). Blue is Dapi signal and indicates nuclei.

**Figure 4:**
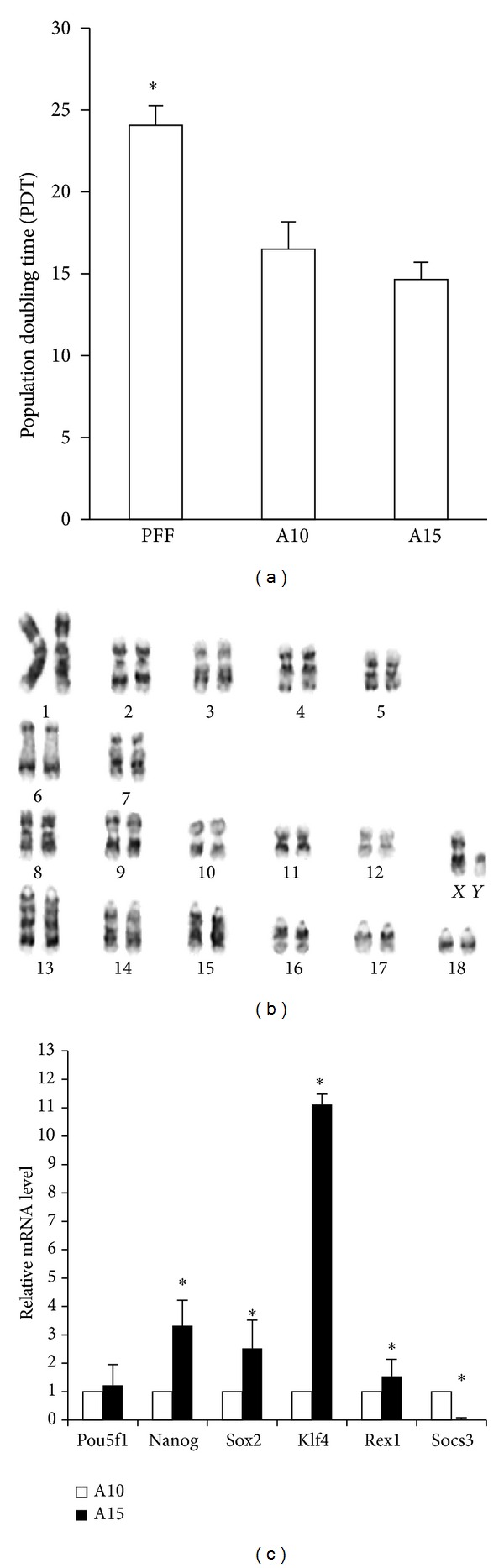
Cytogenetic properties and stem cell marker gene expression analysis in porcine iPSCs. (a) The PDT of both lines (at passage 6) and PEFs were analyzed. (b) Karyotyping indicates a normal chromosomal content in A15 at passage 17. (c) Relative mRNA level of *Pou5f1, Nanog, Sox2, Klf4, Rex1,* and *Socs3* between A10 and A15. *P < 0.05.

 To confirm the characteristics of the porcine iPSCs, the expression stem cell marker, PDT was used and karyotypes performed. Genomic PCR revealed an integration pattern of human transcription factors in both porcine iPSC lines, A10 and A15 (Figure 2). Both lines had integrated all six transcription factors. In addition, the exogenous pluripotent gene expression was identical and showed an integrated pattern. Furthermore, all six pluripotent genes were endogenously expressed in both lines at passage 20. Immunocytochemical analysis confirmed that the cells within the colonies expressed the pluripotency markers in their nuclei and on their surface. Both porcine iPSC lines, A10 and A15, were positive for Oct4, Nanog, and Ssea1 (Figure 3); however, other surface markers, Tra-1-60, Tra-1 81, Ssea3, and Ssea4, were not observed (data not shown). The population doubling time for the porcine iPSCs was approximately 15 h, which was shorter than that of parental PEFs (Figure 4(a)). The karyotyping results showed that porcine iPSCs, after 17 passages, had a normal karyotype of 38 chromosomes with no aneuploidy, tetraploidy, or other visible abnormalities (Figure 4(b)). The RT-PCR results showed that the pluripotency genes in both porcine iPSC lines were constantly expressed in their expansion cultures (until passage 76). However, the expression level of the pluripotency genes of the A10 line was significantly lower than those of the A15 line (*P* < 0.05), except for cytokine signaling-3 (*Socs3*) (*P* < 0.05) (Figure 4(c)).

### 3.3. *In Vitro* and *In Vivo* Differentiation of Porcine iPSCs

**Figure 5:**
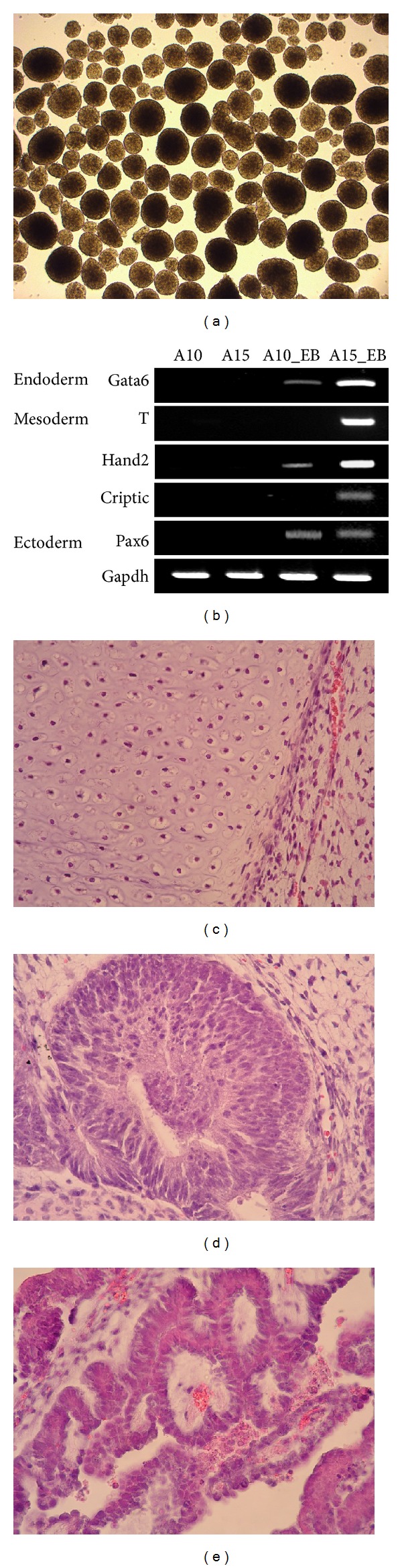
*In vitro* and *in vivo* differentiation of porcine iPSCs. (a) Embryoid bodies were cultured in stem cell media without LIF and bFGF for 10 days. (b) RT-PCR analysis shows that all differentiation makers for the three germ layers were expressed in the EBs of A15 and A10, but mesoderm markers, T (*brachyury*) and *Criptic*, were not expressed in those of A10. To test the *in vivo* differentiation of porcine iPSCs, A10 and A15 lines were injected subcutaneously into a nude mouse. Only the A15 cell line formed teratomas, which were dissected 9 weeks after injection. Hematoxylin-eosin staining shows three types of tissues: (c) mesoderm, cartilage tissue; (d) ectoderm, neuronal tissue; (e) endoderm, glandular epithelium.

 Pluripotent stem cells, which include embryonic stem cells (ES cells) and induced pluripotent stem cells, have the capacity to differentiate into all three germ layers. To test the *in vitro* differentiation ability of porcine iPSCs, they were aggregated using an AggreWell plate and further cultured in stem cell media without bFGF and LIF. Both A10 and A15 cell lines were able to form EBs (Figure 5(a)); however, the A10 cell line showed a limited ability to differentiate. All markers for the three germ layers (ectoderm: *Foxj3* and *Pax6*, mesoderm: *Hand2* and *Criptic*, and endoderm: *Sox17* and *Gata6*) were expressed in the A15 line; however, *Hand2* and *Criptic*, both mesoderm markers, were not expressed in the A10 cell line (Figure 5(b)). A teratoma formation experiment was performed to confirm *in vivo* differentiation. Porcine iPSCs were injected into immune compromised mice to form teratomas. Only the A15 cell line formed teratomas and differentiated into the three germ layers. Nine weeks after injection, the mice were sacrificed and teratomas were collected for histological analysis, which revealed that these teratomas contained three types of tissues: mesoderm, cartilage tissue (Figure 5(c)); ectoderm, neuronal tissue (Figure 5(d)); and endoderm, glandular epithelium (Figure 5(e)). 

### 3.4. Maintenance of Porcine iPSCs

**Figure 6:**
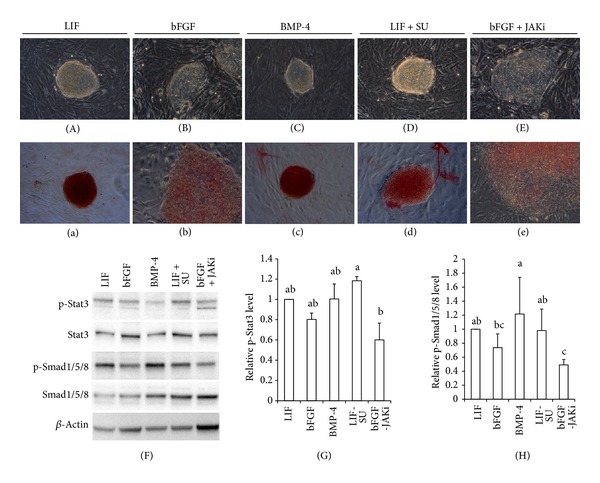
Morphologies and protein expression patterns of porcine iPSCs cultured in various conditions. The A15 line is cultured in medium containing (A) 20 ng/mL LIF, (B) 20 ng/mL bFGF, (C) 20 ng/mL BMP-4, (D) 20 ng/mL LIF and + 2 *μ*M SU, or (E) 20 ng/mL bFGF and 1 *μ*M JAKi, for 48 h. ((a)–(e)) All colonies are positive for AP, regardless of their culture conditions. (F) Western blot shows the phosphorylation levels of Stat3 and Smad1/5/8 proteins in A15 line cultured with different conditions, which were quantitated using ImageJ program ((G), (H)). ^a, b, c^ *P* < 0.05.

 Porcine iPSCs were generated using a combination of transcription factors under stem cell culture conditions, which included 10% KSR and 10% FBS in stem cell media supplemented with bFGF and LIF. To confirm dependency of these iPSCs, they were separately cultured in a stem cell medium supplemented with (i) 20 ng/mL LIF, (ii) 20 ng/mL bFGF, (iii) 20 ng/mL BMP-4, (iv) 20 ng/mL LIF and 2 *μ*M SU, or (v) 20 ng/mL bFGF and 1 *μ*M JAKi, for 48 h (Figures 6(A)–6(E)). For the bFGF treated groups (Figures 6(B) and 6(E)), the colonies showed signs of differentiation and a dispersing colony morphology while losing alkaline phosphatase activity (Figures 6(b) and 6(e)); inhibition of FGF signaling, by the tyrosine kinase inhibitor, Su5402, had no deleterious effects on sustaining the pluripotency of the porcine iPSCs (Figures 6(D) and 6(d)). Furthermore, there were colonies that expanded with tight morphology and showed strong AP activity in the LIF and BMP-4 treated groups (Figures 6(a), 6(c), and 6(d)). Western blotting showed the phosphorylation level of the Stat3 and Smad1/5/8 proteins among the cultured groups (Figure 6(F)). In addition, phospho-Stat3 and phospho-Smad1/5/8 were significantly suppressed by JAKi treatment (*P* < 0.05) (Figures 6(G) and 6(H)).

## 4. Discussion

 Pigs have immunologically and physiologically very similar organs to humans, and their average lifespan is over 20 years, which render them attractive as the sources of clinical models. Recently, germline chimeras as well as cloning pigs were successfully generated using porcine iPSCs [20–22]. Hence, porcine iPSCs could be useful to apply for the generation of disease models, surrogate organs compatible with the human immune system and cloning. Human iPSCs are without doubt powerful cell resources for developmental research and clinical application. While the promise of human iPSCs is great, animal model is essential to test transplantation therapies with iPSCs for safety and efficacy, before the transplantation is applied to human. So far, mice offer as an unrivalled tool for understanding about reprogramming machinery and improving methodology in the field of stem cell based therapy, but their size, physiology, and reduced lifespan make them inadequate as an animal model to test for safety and efficacy of therapy. 

There are several publications describing the generation of iPSC lines from porcine somatic cells [4–6, 21, 23]. Under the four-factor system, naïve-like porcine PSCs were generated, and they could contribute to the fetal development, but low chimerism efficiency (1 out of 13 fetuses) and no germline transmission were observed [23]. In contrast, West et al. [21, 22] reported that piPSCs reprogrammed by human six factors could generate a germline chimera with high efficiency (85.3%). It is likely that four-factor system may be insufficient to reprogram somatic cells into *bona fide* iPSCs in pigs so far. Therefore, we used six factors to generate iPSCs from the Massachusetts General Hospital (MGH) Major Histocompatibility Complex (MHC) inbred miniature pig (MGH pig) and identified their properties. 

In this study, iPSCs were established from pig ear fibroblasts by transduction using lentiviral vectors expressing six human factors: *POU5F1, NANOG, SOX2, C-MYC, KLF4, *and *LIN28*. They were characterized by their morphology, pluripotent gene expression, *in vivo* and *in vitro* differentiation, and cytokine dependency. Two porcine iPSC lines, A10 and A15, were selected for expansion culture and further experiments. A15 grew rapidly as compact, tight colonies and had a dome-shaped appearance, while A10 exhibited a flat but tightly packed morphology. The cells in both lines had a high nucleus/cytoplasm ratio and prominent nucleoli, which is one of the distinct characteristics of PSCs. Both lines were capable of maintaining their pluripotency during continuous culture, but the A10 line had a limited ability to differentiate into all three germ layers both *in vitro* and *in vivo*. Teratoma formation was observed only in the A15 cell line when they were injected into nude mice. Although the A10 cell line had integrated all six transcription factor genes and was expressed exogenously with traditional stem cell marker expression, it failed to form a teratoma. Therefore, these findings suggested that the A10 line either had partially reprogrammed or fully reprogrammed similar to the A15 cell line but differentiated during early culture processing. To understand the differences between the two lines, quantitative analysis of the endogenous expression level of key transcription factor genes was performed using real-time PCR. The expression pattern of the analyzed genes, in the A15 cell line, could be considered as a normal rather than asymmetric pattern by up- or downregulation of exo- or endogenous core genes; this is because, although there were differentiation features observed in the culture of the A15 cell line, it did not exceed similar features observed in the A10 cell line.

 Stem cells express a core group of genes, Oct4, Nanog, and and Sox2, known to core transcription factors as playing key roles in maintaining ESC self-renewal and pluripotency [24–26]. Thus, an asymmetric expression level of these core genes could negatively affect the pluripotent potential of reprogrammed cells. Up- or downregulation of Oct4 or Sox2 leads to divergent developmental fate of the ESCs [27]. The endogenous *Oct4* expression level was not different in comparisons between the two cell lines; however, the *Nanog, Sox2*, and *Klf4* expression in the A10 cell line was significantly lower than the expression in the A15 cell line. Thus, overexpression of *Nanog* or *Klf4* is capable of maintaining the pluripotency and self-renewing characteristics of ESCs [28, 29]. Interestingly, when mESCs were exposed to EpiSC culture conditions, they displayed ES cell-specific marker expression of the primed PSC rather than that of the naïve state, with downregulated *Nanog*, *Klf4,* and *Rex1*, while maintaining *Oct4* expression [17, 29]. This result is consistent with the expression patterns of the same genes in the A10 cell line when compared to the cells in the A15 line and implies that a pluripotent state, among the cells in the A10 line, was more like the primed state than the naïve state. By contrast, one of the highly expressed genes in the A15 line not in the A10 line, was *Rex1* regulated by Sox2 and Nanog cooperation, which suggests that the self-renewal capacity of the A15 line could be increased by high expression of *Sox2* and *Nanog* [30]. However, the expression level of each core gene needed to maintain pluripotency of porcine iPSCs remains unclear; however, the results of the present study suggest that a higher expression level of these core genes in the A15 cell line, but not the A10 cell line, is needed to maintain the naïve state of ESCs. 

 Ssea-1 is expressed in the ICM of mouse embryos, whereas it is not expressed in human embryos [31]. Therefore, Ssea-1 might be a candidate marker for naïve PSCs. Although Ssea1 was expressed in the cytoplasm and on the cell surface of the ICM in porcine embryos [32], its expression in generated porcine iPSCs is not clear. In an initial experiment, Wu et al. [4] and Esteban et al. [6] reported that porcine iPSCs Ssea-3 and Ssea-4, but not Ssea-1, resembled primed PSCs, whereas Ezashi et al. [5] established Ssea-1 and-4 positive porcine iPSCs, but not Ssea-3. In the present study, both iPSCs expressed a surface marker, Ssea-1, but lacked Ssea-3 and -4 in the naïve PSCs. Therefore, subsequently growth factor dependence was examined to identify whether the A15 line more closely resembles the naïve or primed state.

 Mouse and human ESCs are biologically different despite sharing a core genetic regulatory network for pluripotency [33]. One important difference is the dependent signaling for maintaining pluripotency and self-renewal. Mouse ESCs rely on LIF and BMP for self-renewal and pluripotency, while LIF is dispensable in hESCs. By contrast, FGF2 and activin A are the primary determinants of hESC self-renewal and pluripotency. To confirm dependency of the porcine iPSCs, for the A15 cell line, the cells were cultured separately in either LIF or bFGF supplemented conditions. When the porcine iPSCs were exposed to bFGF or the inhibition of LIF by the JAKi, colonies were dispersed and expressed abated AP activity. By contrast, the porcine iPSCs cultured in stem cell media with LIF or BMP-4 were tightly expanded and showed strong AP activity. Indeed, the phospho-Stat3 and phospho-Smad1/5/8 were significantly suppressed by bFGF and JAKi. These findings demonstrate that the A15 cell line was dependent on JAK-Stat3 signaling for continued self-renewal and was not affected by FGF withdrawal and inhibition by the FGF receptor.

 In conclusion, we have generated the porcine iPSCs from the Massachusetts General Hospital (MGH) Major Histocompatibility Complex (MHC) inbred miniature pig (MGH pig), which was established for organ transplantation studies. Because of MHC class I genes encoding critical molecules for delayed xenorejection, the MGH pig is widely used for research in this area as a universal donor for human xenotransplantation. Furthermore, we confirmed that porcine iPSCs established under mouse ESC culture conditions without the addition of any small molecules had characteristics of putative naïve ESCs; they sustain self-renewal and pluripotency and the expression pattern of conventional stem cell markers. Therefore, our porcine iPSCs could offer distinct advantages over other strains as a cell resource for medical and agricultural research and application.

## Figures and Tables

**Table 1:** Primer sets for RT- and real-time qPCR.

Gene name	Primer Sets (5′→3′)		References
	Forward	Reverse	
For RT-PCR*			
* hPOU5F1 *	GATCAAGCAGCGACTATGCA	TCTGGGATGGAAACTGGAA	Ezashi et al. 2009 [5]
* hKLF4 *	CTGCGGCAAAACCTACACAA		Ezashi et al. 2009 [5]
* hNANOG *	CCACTAGGTATTTTAGTACTCC		NM_024865
* hSOX2 *	CCTGGCATGGCTCTTGGC		Ezashi et al. 2009 [5]
* hC-MYC *	GATTCTCTGCTCTCCTCGACG		Ezashi et al. 2009 [5]
* hLIN28 *	GCGGCCAAAAGGAAAGAGCA		NM_024674
* Gapdh *	CTCAACGACCACTTCGTCAA	TCTGGGATGGAAACTGGAAG	X94251
* pPou5f1 *	ACAAGGAGAAGCTGGAGCCG	CGCGGACCACATCCTTCTCT	NM001113060
* pKlf4 *	TGGGCAAGTTTGTGTTGAAG	AGGAAGGGTGGGTAGTTTGG	DQ000310.1
* pNanog *	TGAGGTTTATGGGCCTGAAG	ATTTCATTCGCTGGTTCTGG	NM_001129971.1
* pSox2 *	CAAGATGCACAACTCGGAGA	TGCTGTAGCTGCAGTTGCTC	NM_001123197.1
* pc-Myc *	CAGATCAGCAACAACCGAAA	TCCAACTCTGGGATCTGGTC	FJ882404.1
* pLin28 *	TGCACCAGAGTAAGCTGCAC	CTGCATATTCTTCCCCTTGG	HM347046.1
For RT-qPCR			
* Pou5f1 *	AGCGCTTCAGAAAGATCTCG	GAGCTGCAAAGCCTCAAAAC	NM001113060
* Nanog *	GATTGGGGTGGTTAGCTCCT	TGAAGGTGAGACTCGCTCTG	NM_001129971.1
* Sox2 *	CAGGAGGGAAGACTCCATCA	CTCCCTCTTGGACAGTCGAG	NM_001123197.1
* Klf4 *	GCCCTTAGAGGCCCACTT	GCAGGGCAGGATGACAGT	DQ000310.1
* Rex1 *	AGCTAACCCTGTCCACATCG	CAAGTCAGCAGCAGTCTCCA	XM_003123016.2
* Socs3 *	CTCCGACTGAACCCTCCTC	CGTTGACTGTTTTCCGACAG	HM045422.1

*The reverse primer used for exogenous human *POU5F1*, *KLF4*, *NANOG*, *SOX2*, *C-MYC*, and *LIN28* expression is a part of woodchuck hepatitis virus posttranscriptional regulatory element (WPRE) region within viPS lentiviral vector. h: human; p: pig; F: forward; R: reverse.
